# Aptamer Chimeras for Therapeutic Delivery: The Challenging Perspectives

**DOI:** 10.3390/genes9110529

**Published:** 2018-10-31

**Authors:** Carla Lucia Esposito, Silvia Catuogno, Gerolama Condorelli, Paola Ungaro, Vittorio de Franciscis

**Affiliations:** 1 Istituto di Endocrinologia ed Oncologia Sperimentale “G. Salvatore”, Consiglio Nazionale delle Ricerche (CNR), 80145 Naples, Italy; c.esposito@ieos.cnr.it (C.L.E.); s.catuogno@ieos.cnr.it (S.C.); pungaro@ieos.cnr.it (P.U.); 2 Department of Molecular Medicine and Medical Biotechnology, “Federico II” University of Naples, 80145 Naples, Italy; gecondor@unina.it

**Keywords:** aptamer, chimeras, targeted delivery

## Abstract

Nucleic acid-based aptamers have emerged as efficient delivery carriers of therapeutics. Thanks to their unique features, they can be, to date, considered one of the best targeting moieties, allowing the specific recognition of diseased cells and avoiding unwanted off-target effects on healthy tissues. In this review, we revise the most recent contributes on bispecific and multifunctional aptamer therapeutic chimeras. We will discuss key examples of aptamer-mediated delivery of nucleic acid and peptide-based therapeutics underlying their great potentiality and versatility. Achieved objectives and challenges will be highlighted as well.

## 1. Introduction

Aptamers are small single-stranded oligonucleotides (SSOs) that interact with target molecules because of their unique three-dimensional structures that confer high binding affinity and specificity. Compared to monoclonal antibodies (mAb), aptamers have the advantage to show low or no immunogenicity and toxicity, revealing them as valid and even superior alternatives [1,2,3]. In addition, aptamers have many manufacturing advantages, including simple production, lower costs, low batch-to-batch variation and longer storage timing. Aptamers are selected in vitro by a combinatorial chemistry method named SELEX (Systematic Evolution of Ligands by Exponential enrichment) [4,5]. The SELEX technology is an iterative procedure allowing the selection of aptamers to be able to bind to specific targets of different nature: from small molecules to proteins. The starting point is the synthesis of a single-stranded nucleic acid (DNA, RNA or modified RNA) library characterized by a high complexity and randomization of sequences in order to create a wide sequence diversity. The procedure includes six different steps: (1) the counter-selection step, in which the library is incubated with non-target molecules; (2) the positive selection step, in which unbound aptamers from the previous step are incubated with the target molecules; (3) partition of the unbound aptamers from those bound to the targets; (4) dissociation of the aptamer-target complexes; (5) Polymerase chain reaction (PCR) amplification of the aptamer pool enriched for specific ligands; (6) sequencing to identify the best binders.

Once isolated, aptamers can be manufactured by chemical synthesis and optimized by introducing a wide range of chemical modifications to further improve their pharmacokinetics or pharmacodynamics profile [6]. Since their first description in 1990 [4,5], many aptamers against disease-relevant targets have been selected and employed as valuable tools for therapy and diagnosis [7]. Although the targets of most therapeutic aptamers are extracellular, the finding that aptamers against cell surface molecules can be selectively internalized into target cells permitted researchers to use them as targeting moieties for drug delivery [8]. The design of drug-delivery carriers remains a demanding challenge for the development of safer therapeutic strategies able to only target diseased cells reducing toxicity and undesired effects on healthy tissues in weakened patients. To achieve this goal, several approaches of conjugation of therapeutic molecules with ligands enabling the active binding to the target cells have been proposed [8,9]. Among them, the use of aptamers as delivery carriers has emerged in the last decades, showing several advantages over alternative targeting agents, including mAbs and peptides. Their high affinity and specificity, combined with their easily modifiable chemical nature and the possibility to be potentially generated against targets of different nature, have supported the development of different aptamer-based conjugates for the targeted delivery of secondary reagents. Thus far, aptamers have been functionalized to make them capable of further conjugation with different molecules, including chemotherapy agents, small interfering ribonucleic acid (siRNAs), microRNAs (miRNAs), peptides or even another aptamer. Such hybrid molecules, also termed aptamer-chimeras, are able to specifically bind to target cells and specifically deliver their cargo [10]. In this review, we revise the most recent attempts on bispecific and multifunctional aptamer therapeutic chimeras. We discuss some innovative examples reported in the last few years underlying aptamer potential and challenges and the new directions within the fields.

## 2. Aptamer-si-miRNA Chimeras—An Update

Therapeutic oligonucleotides (ONTs) such as siRNAs, miRNAs, guide RNAs (gRNAs) and antisense oligonucleotides, represent an emerging class of very promising drug molecules. ONTs have the double advantage of being highly specific in target recognition owing to base pairs complementarity and of acting by modulating the expression of disease-associated key genes. However, the use of systemic administration of ONT therapeutics is limited by the presence in the organism of several functional and physical obstacles, as, for example, the anionic charge of oligonucleotides that impedes them to cross the lipid bilayers, and thus, to penetrate into the cells. Various adjuvants have been tested to shield the anionic charges to improve uptake into target cells. An attractive alternative to reach cell-specificity is their conjugation with a ligand that enables active binding to the cell-surface protein on the target cell. Aptamers are able to bind to their cognate targets with high affinity and specificity and may be rapidly internalized in a receptor-mediated manner, thus have been largely used to generate chimeric molecules for the delivery to a designated cell of siRNAs and miRNAs [11].

**Figure 1:**
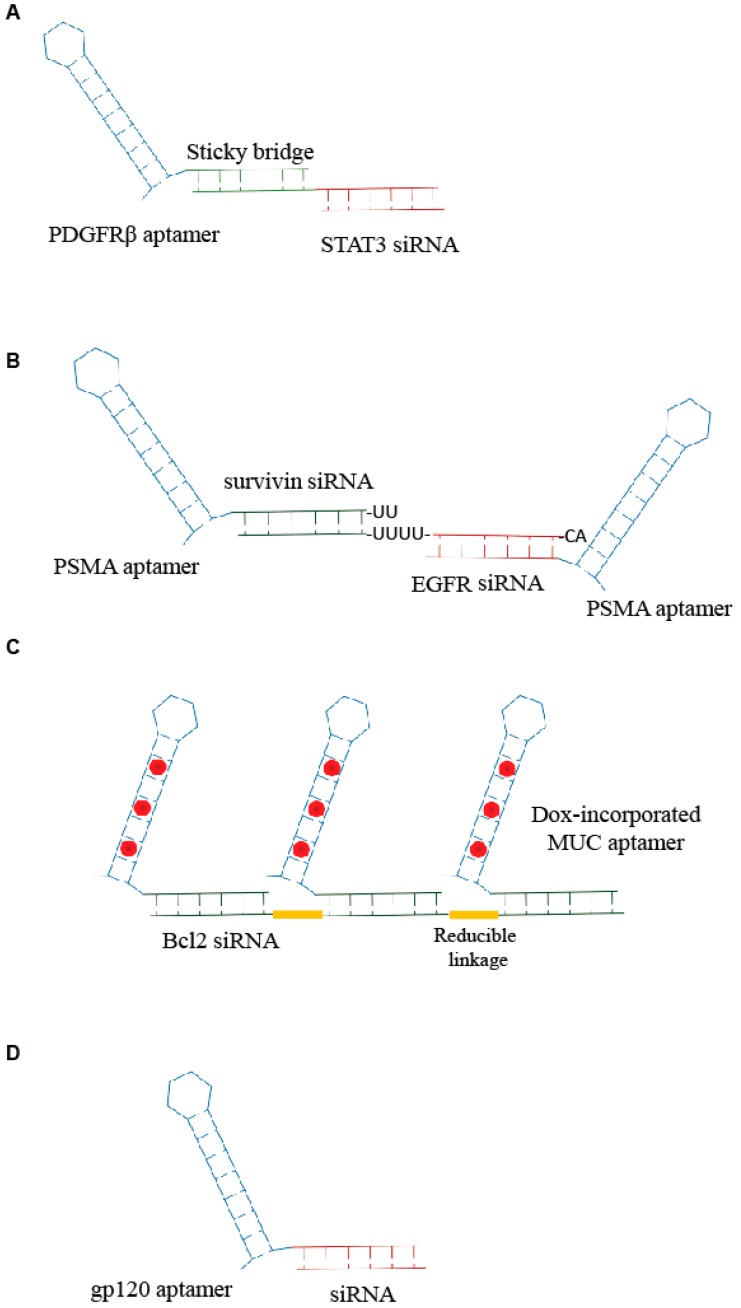
Scheme of examples of aptamer-small interfering ribonucleic acid (siRNA) conjugates. (**A**) Sticky-based approach developed by Esposito et al. [11]. (**B**,**C**) Example of multimeric structure developed for siRNA delivery [16,17]. (**D**) Unique RNA molecule developed for aptamer-mediated delivery of a promoter-targeted small RNA [7].

Combinations of aptamers and siRNAs have been referred to as AsiCs or aptamer-siRNA conjugates. The first generation of AsiCs was the conjugate of the prostate-specific membrane antigen, PSMA, Aptamer (A10) with polo-like kinase 1 (Plk1) or Bcl2 siRNA, A10-Plk1 and A10-Bcl2 siRNA [12]. Thus far, a number of chimeras have been developed for targeted delivery of siRNAs in order to suppress the overexpressed genes involved in several pathologies, including cancer [13]. Very recently, Esposito et al. [11] have published the use of an aptamer-siRNA chimera (Gint4.T-STAT3) that binds to the oncogenic receptor platelet-derived growth factor receptor-β (PDGFRβ), Gint4.T, and specifically, target STAT3 into glioblastoma (GBM)-derived cells in a receptor-dependent manner. The aptamer-siRNA chimera was generated by using a stick-based strategy [14] in which the aptamer and the siRNA are linked through the presence of complementary 17 bp sequences (Figure 1A). The developed AsiC was able to reduce cell viability and migration in vitro and to inhibit tumor growth and angiogenesis in vivo in a subcutaneous xenograft mouse model, proving to be a molecule with a great translational potential for GBM therapy.

Further developments of the aptamer-based siRNA delivery strategies involves the generation of multimeric structures. An innovative example comes from Liu et al. [15]. The authors developed an RNA-based bivalent aptamer-dual siRNA chimera, named PSMA aptamer-Survivin siRNA-EGFR siRNA-PSMA aptamer (PSEP), in which two PSMA aptamers were joined together spaced by two different siRNAs: one specific for the epidermal growth factor receptor (EGFR) and the other for survivin, a member of the inhibitor of apoptosis family, IAP. The PSMA aptamer-survivin siRNA-EGFR siRNA-PSMA aptamer chimera was able to inhibit EGFR and survivin simultaneously and induce apoptosis in vitro and in vivo suppressing both tumor growth and angiogenesis. Similarly, Xue et al. [16] described a chimera in which the anti-EGFR siRNA was fused between two HER2 aptamers (Figure 1B). The bivalent conjugate effectively down regulated EGFR and HER2 synergistically, inducing apoptosis both in vitro and in vivo in breast cancer xenograft models. 

Another multivalent aptamer-siRNA conjugate was described by Jeong et al. [17]. They generated a chimera containing multiple mucin-1 (MUC1) aptamers conjugated to anti-Bcl2-siRNA and loaded with doxorubicin (Dox), an anticancer drug that is able to intercalate within nucleic acids. Aptamers and siRNA were crosslinked through reducible linkages, and the Dox was incorporated within the generated aptamer-siRNA (Figure 1C). The conjugate that resulted was able to induce an efficient anticancer action on drug-resistant cancer cells. 

Recently, Alshaer et al. [18] described an aptamer-based siRNA delivery system employing an anti-CD44 aptamer and an anti-Luciferase 2 (Luc2) siRNA condensed with non-cationic protamine peptide in order to allow the loading into polyethylene glycol (PEG) conjugated liposomes. The authors demonstrated that the system gave an efficient silencing of the reporter gene in a CD44-positive triple-negative breast cancer model. The study provides a proof of principle of a novel aptamer-based targeting strategy to achieve an effective and specific silencing in cancer. 

Apart from cancer, aptamer-mediated strategies have been developed for various pathologies, including transmissible diseases such as human immunodeficiency virus (HIV) infection [19]. Basing on their previous studies on aptamer-mediated delivery of anti-HIV-1 siRNAs [14,20,21], Zhou J. et al. [22] recently addressed the delivery of interfering RNAs directed against gene’s promoter. These sequences cause epigenetic modifications (i.e., histone and DNA methylation) that result in effective and long lasting transcriptional gene silencing (TGS). The authors covalently conjugated anti-HIV gp120 aptamer to one small RNA, LTR-362 (Figure 1D), targeting HIV-1 5′ long terminal repeat (LTR) inducing TGS. They demonstrated functional delivery and conjugate-mediated TGS in HIV-1 both in vitro and in vivo.

The same group also developed an elegant approach for viral inhibition combining aptamer and shRNA [23]. They selected new aptamers against virus integrase and induced their expression by inserting them in the terminal loop of anti-HIV Tat-Rev shRNA generating a “chimeric” expression construct with a very effective viral inhibition. 

MiRNAs, as another class of short non-coding sequences of nucleotides, are involved in RNAi pathways that can regulate different biological processes. The conjugation of aptamers to miRNA molecule has been addressed in the last years allowing the generation of different chimeras (also referred as AmiCs). 

**Figure 2:**
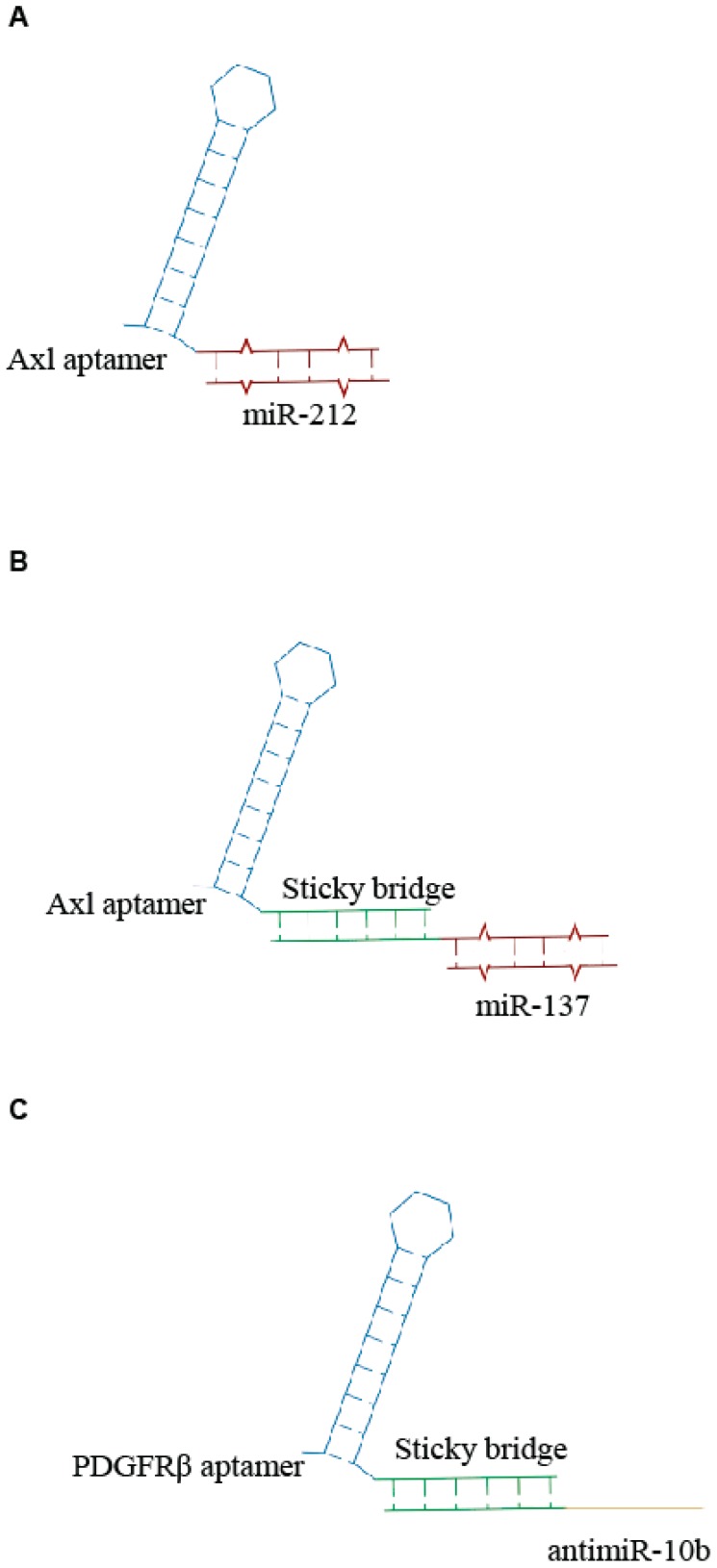
Scheme of examples of aptamer- microRNAs (miRNA) conjugates. (**A**) Unique anti-Axl-miR212 AmiC [24]. (**B**,**C**) Sticky-based approach for conjugation of aptamer to miR (**B**) or antimiR (**C**) [25].

Recently, Iaboni et al. [24] designed an aptamer-miRNA chimera system, consisting of GL21.T, an RNA aptamer recognizing the tyrosine kinase receptor Axl and miR-212 to target non-small-cell lung cancer (NSCLC) cells. The aptamer was covalently linked to the miR-212 sequence, generating a unique RNA molecule (Figure 2A). They demonstrated that the miR-212-GL21.T conjugate could markedly inhibit the anti-apoptotic protein PED/PEA-15 involved in the tumor resistance to chemotherapy treatment. The results showed that this chimera system could increase the caspase activation and reduce the viability of tumor cells in combination with TNF-related apoptosis-inducing ligand (TRAIL) therapy. The same aptamer was also conjugated with miR-137 by a sticky-based strategy (Figure 2B) and used in combination with a second chimera containing the Gint4.T anti PDGFRβ aptamer linked to the antimiR-10b (Figure 2C) for the efficient therapeutic targeting of GBM stem-like cells (GSCs) [25]. When used in combination, the two conjugates synergize to effectively inhibit GSC propagation. Notably the molecules were transported through an in vitro blood-brain barrier (BBB), thus providing a novel combination therapy with great potential for GBM. 

Very recently, we described the use of an additional aptamer- miR chimera (GL21.T/miR 34c), targeting the Axl transmembrane receptor in NSCLC cells. For the conjugation, we employed the sticky-based strategy and demonstrated that the GL21.T/miR-34c AmiC affects NSCLC cell proliferation and is able to overcome acquired receptor tyrosine kinase (RTK)-inhibitor resistance [26].

## 3. Aptamer Bi-Specific and Nanostructures Conjugates—An Update

The chemical nature of aptamers gives the potential to easily engineer them in multivalent structures to achieve different purposes in diagnosis and therapy. 

**Figure 3:**
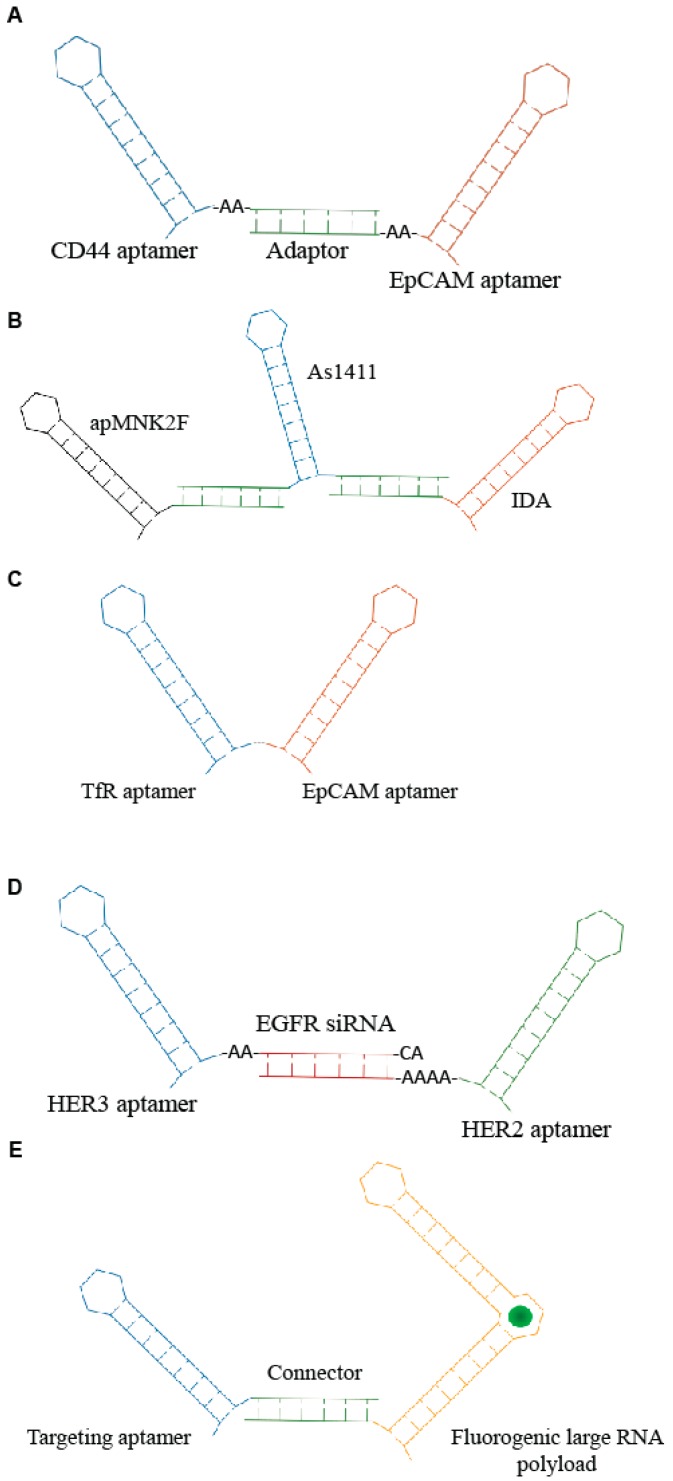
Scheme of examples of multi-modular aptamers. (**A**) Bi-specific molecules developed by Zheng et al. [29]. (**B**) Three aptamers-based conjugate described by Abnous et al. [30]. (**C**) Bi-specific aptamer developed by Macdonald et al. [31]. (**D**) Trivalent molecule generated by Liu group [32] containing anti- HER2 and HER3 aptamers and epidermal growth factor receptor (EGFR) siRNA. (**E**) Aptamer-based modular nanostructure designed by Porcian et al. [33] for the delivery of large RNA payloads.

One of the first applications of multimeric aptamers has been the development of agonistic molecules by linking RNA aptamers against immune-modulating targets, including CD28, CD40, OX40 and 4-1BB. Such molecules have been used to induce an immunogenic response against cancers [27,28]. Subsequently, bi-specific constructs have been developed to allow the simultaneous recognition of multiple targets and the combination of multiple functions. This approach enhances the specificity as well as the efficacy of the molecule. A recent example comes from Zheng et al. [29]. The authors combined two aptamers targeting cancer-relevant epitopes, CD44 and epithelial cell adhesion molecules (EpCAM), respectively, through a double strand adaptor containing an annealed portion of 23 bp and 2–3 unpaired bases (Figure 3A). The resulting RNA molecules inhibited both CD44 and EpCAM, giving a more effective block of cell growth in vitro. Furthermore, the bispecific CD44-EpCAM aptamer inhibited tumor growth in intraperitoneal ovarian cancer xenograft model more effectively then each single aptamer used alone or in combination, showing an improved circulation half-life. Instead, an innovative complex of three therapeutic aptamers has been described by Abnous et al. [30] for prostate and breast cancer therapy. The conjugate combined the DNA aptamers, targeting nucleolin (As1411), integrin α6β4-specific (IDA) and anti- mitogen-activated protein (MAP) kinase interacting kinase 1 (apMNK2F) that were self-assembled through a “stick-based”-like strategy (Figure 3B). The resulting molecule showed enhanced antitumor activity both in vitro and in vivo.

Other multimeric conjugates have been developed for the targeted delivery of functional aptamers and/or siRNAs. For example, Macdonald et al. [31] used the generation of a bi-specific aptamer (Figure 3C) to deliver to the brain a DNA aptamer (EpA) against EpCAM. As delivery moiety, they adopted a second aptamer targeting the transferrin receptor (TfR) that permits the receptor-mediated transcytosis (RMT) of ligands through the BBB. The resulting bifunctional DNA aptamer in which the targeting specificity and sensitivity of both moieties were preserved showed a rapid uptake into the target cells in a receptor-dependent manner. Furthermore, by measuring the trans-endothelial electrical resistance in an in vitro model of BBB, the authors showed that the bifunctional conjugate is capable of penetrating through a monolayer of TfR-expressing endothelial cells and target EpCAM-expressing MDA-MB-231 cells seeded in the lower compartment of the chamber. Most importantly, biodistribution analysis in healthy nonobese diabetic/severe combined immunodeficiency (NOD/SCID) mice indicated that the bifunctional aptamer accumulates in the brain, although penetration at low efficiency of EpCAM non-targeting aptamers in a receptor-independent manner was visible in the brain as well. 

Another interesting recent example underlining the great potential of aptamer complexes has been reported by Liu et al. [32]. They developed a unique molecule consisting of two aptamers and a siRNA for the treatment of drug-resistant HER2-positive breast cancer. In detail, a siRNA against the EGFR was used to connect two aptamers that bind and inhibit HER2 and HER3, respectively, generating a trivalent molecule, termed H2EH3 (Figure 3D). This construct combines the block of HER2/HER3 pathway and silencing of EGFR by the selective delivery of EGFR siRNA, resulting in an effective tumor inhibition in vivo. 

Despite the great advances in the aptamer-mediated delivery of small ONTs such as siRNAs and miRNAs, aptamer use to drive larger RNAs within the target cells remains a challenging goal. One key limit is the necessity to preserve aptamer folding avoiding the interference of the cargoes.

Porciani et al. [33] addressed the delivery of large RNA payloads by designing an aptamer-based modular nanostructure. The modular molecule contained a cell-internalizing aptamer as targeting module and an improved “Broccoli” (dB) aptamer of 176 nt as payload module. Broccoli aptamers [34] are an enhanced version of the spinach family [35], a class of fluorogenic RNA aptamers able to incorporate within their structure molecules resembling the green fluorescent protein (GFP) chromophore. This kind of chromophore gives a high fluorescent signal only after its association with aptamer, thus allowing it to follow the structural integrity and the processing within the target cells. Modular molecules were designed to be employed as targeting modules, either the Waz aptamer targeting TfR [36] or the C10.36 aptamer that is able to recognize B cell cancer cell lines [37]. Targeting and payload modules were assembled via a stick-like based strategy (Figure 3E). The developed structure effectively internalized in a receptor-mediated manner into target cells co-localizing with endosomes and persisting structured for ≥2 h. Dual labeling experiments also showed that the structure remained connected within endosome after 1 h. This work provides the first proof of principle of aptamer ability to drive larger oligos. In addition, it describes a new approach to follow conjugate fate into the target cells. It is accepted that once bound to target cells, aptamer conjugates are internalized through the recycling of the target receptor followed by uptake in the endosomal compartment. However, the mechanism of endosomes escape of the functional cargo still remain elusive and represent a crucial aspect to be deepen for the improvement of the targeted delivery effectiveness. 

## 4. Aptamer-Peptide Conjugates

Short peptides and antibodies have been developed as precise alternatives to conventional therapies for several human diseases, including cancer [38,39]. Despite some encouraging results obtained with cell penetrating peptides (CPPs), their use is not conclusive, owing to the lack of an adequate cell-type specific targeting [40,41]. In this perspective, aptamers may represent very interesting carrier molecules, allowing both selective targeting and effective internalization. Moreover, given to their small size aptamers also show a good tissue penetration that for solid tumors is decisive for the efficacy of therapeutics [42]. For all these peculiar characteristics, aptamers have been recently proposed by different research groups as carrier systems for the delivery of therapeutic peptides. 

**Figure 4:**
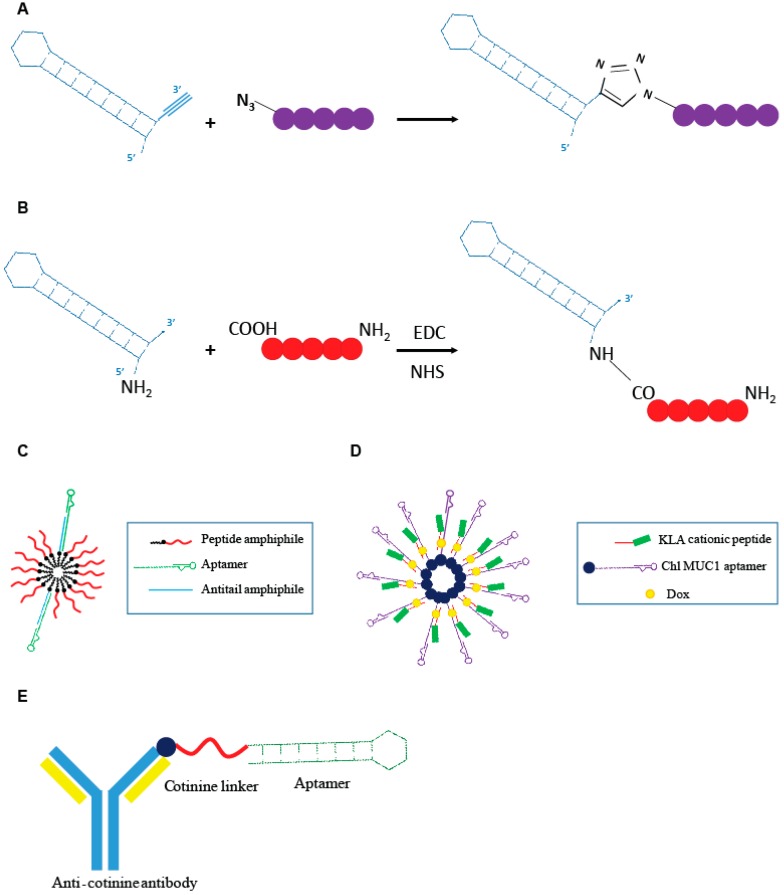
Schematic representation of aptamer-peptide conjugates. (**A**) Direct conjugation through click chemistry of the Gint4.t anti- PDGFRβ aptamer to the active mimetic peptide (MP) described by Romanelli et al. [43]. (**B**) Covalent conjugation of the AS1411 anti-nucleolin aptamer to the cancer cytotoxic melittin peptide described by Rajabnejad et al. [46]. (**C**) Modular peptide amphiphile micelles (PAMs) system functionalized with C10.36, anti-human B leukemia cells aptamer described by Smith et al. [47]. (**D**) Modular DNA micelles system for the co-delivery of doxorubicin (Dox) and KLA cationic pro-apoptotic peptide by using the cholesteryl (Chl) modified anti-multiple mucin-1 (MUC1) aptamer as delivery moiety [48]. (**E**) Aptamer-antibody conjugate, “oligobody”, through the conjugation of the anti-cotinine antibody to the cotinine modified anti- Vascular Endothelial Growth Factor (VEGF) aptamer developed by Heo et al. [50].

Recently Romanelli et al. [43] developed a peptide (R7W-MP) that, by targeting Cavβ2, demonstrated to be able to correct the L-type calcium channel (LTCC) density at the plasma membrane, often hampered in various cardiovascular pathological conditions. The R7W-MP peptide is a fusion molecule composed by a R7W CPP fused to a mimetic peptide (MP), able to target the Tail Interacting Domain (TID) in the Cavβ2, inhibiting its interaction with the C-terminal coiled-coil tail (C-tail) in the site where Cavβ2 is phosphorylated by Akt, and thus, preventing Cavβ2 structural rearrangements responsible for increased LTCC density at the plasma membrane. The R7W-MP peptide revealed to be able to reestablish normal calcium balance and recover myocardial contractility in mouse models of diabetic cardiomyopathy [44]. In order to overcome limitations showed by the R7W-MP peptide, essentially in reference to the lack of selectivity of the R7W moiety, the same group developed an aptamer-based approach for the selective delivery of MP mimetic to cardiac cells [43]. The authors described an innovative approach for direct conjugation of the MP to a 2′-fluoro pyrimidines modified RNA aptamer, named Gint4.T, specifically targeting the PDGFRβ that is abundantly expressed in cardiomyocytes [45]. For direct conjugation of the MP to the aptamer sequence, they proposed a click chemistry approach (Figure 4A) in which all the reactions were performed in DMSO-free conditions and *N*,*N*,*N*′,*N*′,*N*′′-pentamethyldiethylenetriamine was used as a Cu (I) stabilizing agent, in order to prevent RNA degradation caused by Cu (I) disproportionation and subsequent redox reactions. This kind of procedure is, as its main advantages, cost-effective and avoids the use of solvents that may affect the correct aptamer folding. The ability of the Gint4.T-MP chimera to internalize into HL-1 PDGFRβ-expressing cardiac cells, effectively restoring LTCC protein levels; additionally, normal calcium flux was demonstrated in vitro. The study provides the first proof of concept about the use of an aptamer-based system for the selective delivery of a small therapeutic peptide to cardiac cells.

More recently, another example of an aptamer-small peptide conjugate was proposed by Rajabnejad et al. [46]. They generated a chimera in which the anti-nucleolin aptamer (AS1411), used as a carrier moiety, was modified at the 5′ end with a NH2 group and covalently linked to melittin, an amphipathic peptide derived from the honey bee venom with proved cytotoxicity on various cancer cell lines (Figure 4B). Treatment with the AS1411-melittin conjugate revealed to efficiently reduce cell viability in A549 nucleolin-expressing NSCLC cells, with an effect greater than that given by the melittin alone. Importantly, the chimera, unlike unconjugated melittin, demonstrated to poorly affect L929 (nucleolin negative) cell viability, indicating the targeted delivery of the peptide into nucleolin positive cells through RMT. Interestingly, the AS1411-melittin conjugate showed a significantly lower hemolytic activity, which is the major cause of in vivo toxicity, when compared to unconjugated melittin.

In order to further increase the delivery efficacy and the drug bioavailability, some nanoparticle-based aptamer-peptide conjugates have been developed. 

Smith et al. [47] designed an innovative modular peptide amphiphile micelles (PAMs) system functionalized with the G-quadruplex DNA aptamer, C10.36, able to specifically bind and internalize into human B leukemia cells [37]. A DNA oligonucleotide amphiphile with a specific “antitail” at the 3′ end was incorporated into the PAM (A/PAMs) and then the C10.36 aptamer, containing a complementary 3′ tail, was annealed to the amphiphile antitail (Figure 4C). The generated complex preserve the peculiar advantage of using PAM systems, which is its very high drug loading capability, and the selectivity of the delivery given by the aptamer moiety. The authors demonstrated that the C10.36~A/PAMs complex was stable over several hours in serum albumin at levels comparable to that found in blood, as well as in normal cell culture conditions (10% fetal bovine serum, FBS) and in the presence of cell membrane models. Additionally, the specific targeting of human B leukemia cells was proven in vitro. The proposed approach represents a very interesting and flexible technology to improve the delivery of peptide cargoes in terms of bioavailability, selectivity and effectiveness.

Charbgoo et al. [48] developed a system of drug co-delivery aimed to improve anticancer treatment efficacy. They achieved the co-delivery of Dox and KLA cationic pro-apoptotic peptide in tumor cells through an aptamer-based modular DNA micelles system (Figure 4D). The aptamer used was the anti-MUC1 aptamer, able to specifically recognize the MUC1 receptor that is over-expressed in different tumor cell lines. The presence of the cationic peptide on the surface of the DNA micelles were demonstrated to be able to increase resistance to nucleases. The complex remained completely stable over 6 h of incubation in 50% FBS. Aptamer-dependent specific delivery of DNA micelles to MUC1 positive MCF-7 cells was demonstrated in vitro. The conjugate revealed to possess a reduced toxicity and improved efficacy in reducing tumor growth in tumor-bearing mouse models, when compared with free Dox administration.

A similar system of drug co-delivery was developed by Ji et al. [49]. The authors designed a novel DNA nanotube–peptide (DNP) superstructure in which a CPP cRGD (Arg-Gly-Asp-d-Phe-Lys), which can improve accumulation and retention of the molecule by binding to integrin receptors (αvβ3), was covalently linked on the surface of a six-helix structure DNA nanotube. Then, Dox was intercalated into the six-helix DNA structure and Paclitaxel (PTX) infiltrated into the barrel-shaped channel. Three locked DNA strands were used to maintain the complex in a closed state and the anti-MUC1 aptamer, used as selective carrier moiety, was part of one locked DNA strand. The system was also able to function as a sensor system for messenger RNA (mRNA) detection. In the presence of MCF-7 cells, the mRNA one locked DNA strand was designed to be complementary to the target mRNA sequence hybridized with it resulting in the opening of the six-helix-bundle pore channel, and PTX and Dox releasing. The biocomplex was validated both in vitro and in vivo and demonstrated to be able to specifically deliver Dox and PTX in MCF-7 MUC1 positive cells and improved efficacy in reducing cell viability and in vivo tumor growth, when compared to similar biocomplexes loaded with single drugs. Moreover, the biocomplex showed a very low toxicity, a good resistance to nucleases and revealed to be able to effectively detect MCF-7 target mRNA in vitro, and thus, representing an interesting platform for theranostic applications.

In order to combine advantages of nucleic acid aptamers and mAbs [50], Heo et al. developed an aptamer-antibody complex, reported as “oligobody”, as an innovative delivery system. The authors linked an anti-cotinine antibody (cot-body) to the anti-Vascular Endothelial Growth Factor (VEGF) aptamer pegaptanib cotinine modified (cot-pega) (Figure 4E). Cotinine is a metabolite of nicotine that is non-toxic, physiologically absent and pharmacologically inert. The goal is to overcome limitations of using antibodies or aptamers in therapy. Despite their numerous advantages, mAb suffer from some limitations, with the most serious represented by a low tissue penetration that limits their therapeutic efficacy. 

On the other hand, aptamers, owing to their small size have good tissue penetration and persistence; however, they typically have rapid clearance in blood and poor pharmacokinetic profile. The authors demonstrated that the oligobody retains the ability to bind VEGF, with an affinity constant similar to that of cot-pega alone. Interestingly, the half-life of cot-pega was significantly increased in vivo when the oligocomplex was used instead of cot-pega alone. Moreover, the oligobody was revealed to effectively inhibit tumor growth in vivo in NSCLC xenograft mouse models, showing an increased tissue penetration, indicating that aptamer-antibody complexes may represent an efficacious delivery strategy for anti-cancer therapeutics.

## 5. Aptamer Conjugates for the Selective Delivery of Gene Editing Components

In recent years, the development of targeted delivery has progressively increased. A targeted delivery technique should have two indispensable characteristics: a vehicle capable to protect RNA from degradation by nucleases and a ligand delivering cargo into cells or tissues by recognizing a specific receptor. Nucleic acid-based aptamers are folded SSOs having the potential as targeting agents since they bind at high affinity and high specificity their targets. Nevertheless, the potent ability of aptamers as targeting moieties for therapeutic genome editing is still controversial. In the last few years, the clustered regularly interspaced short palindromic repeats (CRISPR) associated protein 9 (Cas9), termed CRISPR-Cas9, is being exploited as an efficient DNA-targeting strategy. This is powered by the addition of the gRNA driving Cas9 to the desired DNA locus for DNA cleavage [51]. Because of its specificity, the CRISPR/Cas9 system has emerged as a powerful and precise tool for the treatment of genetic disorders, cancer and virus infection [52,53]. To date, its clinical applications still remains an unmet goal because of efficient in vivo delivery of CRISPR-Cas9 components [54]. For the CRISPR/Cas9 delivery system to work well, a targeting ligand with high specificity and affinity to a cellular receptor is needed [55]. Conventional approaches to deliver CRISPR-Cas9 system into cells are the use of viral vectors [56,57,58], such as adeno-associated viral (AAV) [59,60,61], adenoviral (Ad) [62,63,64] and lentivirus [17,65], and nonviral vectors such as Lipofectamines [10,66], X-tremeGENE [17], polyethylenimines (PEIs) [58,67], CaP [68] and CPPs [69], which, unlike viral vectors, are safer and show low immunogenicity [58]. In this context, aptamers against cell specific surface receptors have been successfully adapted for the targeted delivery of active therapeutics in vitro and in vivo. An example of well-established and well-characterized aptamers for targeted delivery, is represented by the anti-PSMA aptamers [70]. The targeted delivery of CRISPR/Cas9 system has not been reported yet. Zhen and et al. [71] created an aptamer-liposome-CRISPR/Cas9 chimera incorporating an RNA aptamer that specifically binds to the cell-surface receptor PSMA. Cationic liposomes were linked to aptamers and were used to deliver therapeutic CRISPR/Cas9-gRNA targeting polo-like kinase 1, a pro-survival gene overexpressed in most human tumors, into prostate cancer cells. The aptamer-liposome-CRISPR/Cas9 chimeras obtained showed a specific binding and a remarkable gene silencing effect in vitro; additionally, it induced a conspicuous tumor regression [71]. Another example of tumor cell-targeted delivery of CRISPR/Cas9 by aptamers came from the study of Liang et al., who screened an osteosarcoma (OS) cell-specific aptamer (LC09) and constructed CRISPR/Cas9 plasmids encoding VEGFA gRNA and Cas9. They encapsulated the CRISPR/Cas9 plasmids into a non-virus plasmid, in which the LC09 aptamer was conjugated to PEG-PEI-Cholesterol (PPC) lipopolymer. The LC09 aptamer got efficient delivery of CRISPR/Cas9, leading to effective VEGFA genome editing in the tumor. The result obtained was the inhibition of orthotopic OS malignancy and lung metastasis, reduced angiogenesis and bone lesion with no detectable toxicity in a syngeneic orthotopic OS mouse model. This study demonstrates that the aptamer-functionalized PPC lipopolymer could be a promising novel CRISPR/Cas9-based genome editing approach for treatment of OS and again show new clinical approaches using CRISPR/cas9 in cancer treatment [72].

Liu et al. constructed dual-targeting polymer/inorganic hybrid nanoparticles to improve genome editing in targeted tumor cells. The CRISPR-Cas9 plasmid for CDK11 knockout was encapsulated in the core of the vector composed of protamine sulfate (PS), calcium carbonate (CaCO_3_) and calcium phosphate (caP), which is decorated by electrostatic interactions to form the dual-targeting delivery system. The AS1411 aptamer, exhibiting high binding affinity for nucleolin [2,73,74], was introduced to the gene delivery system by self-assembly to improve the cell uptake efficiency, as well as to enhance nuclear translocation. The dual-targeting delivery system obtained was capable to deliver the CRISPR-Cas9 plasmid into tumor cells leading to a 90% decrease in CDK11 expression. Cyclin-dependent kinase 11 (CDK11) play an important role in regulating cellular functions such as cell growth and proliferation and its inhibition can induce cancer cell apoptosis and reduce migration and invasion activities [75,76].

Currently, non-viral delivery of preassembled CRISPR ribonucleoproteins have been developed for somatic gene-editing applications [77,78]. However, variable delivery of the CRISPR system includes imprecise insertions and deletions. In order to promote a more faithful writing of the human genome, some authors combined a short RNA aptamer with streptavidin, termed S1mplex, to complex CRISPR-Cas9 ribonucleoproteins with a nucleic acid donor template. In addition, the complexes are enriched for edited cells with additional moieties such as fluorophores or quantum dots. In human cells, tailored S1mplexes enrich the ratio of precisely edited to imprecisely edited alleles up to 18-fold higher respect to standard gene-editing methods. Cell populations containing multiplexed precise edits enriched up to 42-fold. The S1mplex strategy provides a straightforward tool to regulate gene-editing applications in vitro and potentially in vivo [79]. 

## 6. Aptamer In Vivo Administration

For a restricted number of pathologies affecting easily accessible tissues, such as eyes, skin or muscles, the local administration of aptamers represents a valid and effective approach. This way of administration shows the double advantage to reduce the administered dose, and thus, reducing the overall toxicity.

For the majority of diseases, the target tissues are less accessible and systemic administration is needed. In this case, for the success of the therapy, different obstacles need to be overcome. Before reaching their target sites, aptamers need to cross many physical barriers into the organism, resisting to serum nuclease degradation and to phagocytosis by the reticuloendothelial system, and to avoid massive renal secretion. Aptamers, in most cases, lack of a depth description about their pharmacokinetics (stability and biodistribution) and pharmacodynamics (toxicity and immunogenicity), as more comprehensive in vivo studies are necessary. 

Nevertheless, many aspects have been addressed by the use of different modifications that can be easily introduced within the aptamer sequence. Commonly, aptamers are modified at the 2′ position with fluoro- (F), amino- (NH_2_) or O-methyl (OCH_3_) groups, or by using locked nucleic acids (LNA). These modifications greatly improve the resistance to serum nucleases, and additionally, can be directly incorporated during the selection process [80,81,82], avoiding the risk to alter aptamer folding necessary for specific binding when included post-SELEX.

A very interesting class of molecules is represented by the so called SOMAmers (low Off-rate Modified Aptamer, by SomaLogic, Boulder, CO, USA). They are aptamers containing deoxyuracils modified with hydrophobic groups at the positon C5 that highly increase target affinity and stability [83,84]. Additionaly, Spiegelmers are very interesting molecules. They are chiral L-form RNA aptamers not recognized by nucleases and showing enhanced in vivo stability [85]. Despite neither SOMAmers nor Spiegelmers having been employed in targeted delivery thus far, they underline the great repertoire of possibility offered by aptamer modifications well. 

Given their very small size (6–30 kDa), aptamers are rapidly excreted through renal filtration following intravenous injection. To overcome this problem, they can be conjugated with different molecules, such as PEG [82], cholesterol [86,87], proteins [50,88], liposomes [89], organic or inorganic nanomaterials [90,91] in order to increase aptamer molecular weight, and thus, prolonging their circulation time. For example, PEGylation of Macugen, the anti-VEGF aptamer approved by the FDA for the treatment of wet age-related macular degeneration, increased half-life to 9.3 h and 12 h in plasma after IV injection or subcutaneous injection, respectively [92,93]. 

Despite the advantages in terms of improved performance and extended circulation time, aptamer formulation can be associated with occurrence of adverse effects. For example, allergic responses have been observed with PEG in a phase III study of the aptamer-based anticoagulation system, REG1 (Regado Biosciences, Bernards, NJ, USA) [94]. Moreover, in general lipophilic molecules may result in aspecific liver accumulation and hepatotoxicity [95]. Hence, prudent formulations are necessary for efficacy and safety of aptamer therapeutics.

## 7. Targeting the Brain

Because of the high precision of the intracellular target recognition RNA-based drugs are emerging as very promising for targeted gene replacement or editing representing a concrete hope to manage devastating brain pathologies including genetic brain disorder, those related to protein aggregation (Alzheimer’s, Parkinson’s and prions) and brain cancers. For effective systemic treatments, naked oligonucleotides require appropriate pharmacokinetic and pharmacodynamic profiles while preserving their ability to reach the active intracellular sites and modulate gene expression. However, an additional impediment to the development of gene therapeutic compounds for the central nervous system (CNS) is the presence of the tight physical and physiological barriers that protect the brain from infectious and toxic agents [96]. The tight cerebrovascular endothelium named BBB is a cellular barrier composed of brain capillary endothelial cells, pericytes and astrocytes that regulates the ionic composition for proper synaptic signaling function, and additionally, impedes the passive transport of large macromolecules, preventing researchers to develop targeted treatments with ONTs and to investigate on the functional processes in the CNS. 

Two decades ago, Pardridge et al. first addressed the active transport of large therapeutic molecules through the BBB by taking advantage of the RMT mechanism of transport. To this end, by using as carrier a mAb against an endogenous BBB receptor transporter, such as the insulin or TfRs that are highly expressed on endothelial cells [97,98]. The receptor-specific mAb penetrates the brain by receptor-mediated endocytosis acting as a “Trojan horse” to deliver therapeutic cargoes into the brain. As targeting moieties for cell surface receptors, aptamers hold specific recognition of molecular biomarkers of the affected cells and have advantages compared to antibodies, such as low immunogenicity and superior tissue penetration [99]. 

In 2011, Holahan et al. [100] addressed the use of aptamers to directly target brain functions in vivo. In order to overcome the impediments to transport through the BBB, the authors injected a dopamine-binding DNA aptamer, termed DBA, into the nucleus accumbens of the rat brain showing its ability to regulate NMDA-receptor antagonist-induced cognitive deficits indicating that the aptamer effectively bound in vivo to dopamine.

Since then, an important step has been the generation of RNA aptamers able to penetrate an intact health barrier. A few years ago, Cheng et al. [101] conceived a novel in vivo SELEX protocol injecting the library into the peripheral vasculature and using as target the brain in living wild-type mice. To this end, they used a high complexity starting library (with the random region of 40 nt) consisting of 2′-fluoropyrimidine–modified RNA to enhance nuclease resistance. They performed 22 SELEX rounds allowing the circulation of the library for either 1 h or 3 h, at each round, brains were harvested and aptamers recovered. The authors determined whether specific aptamers could be identified that would enter the brain. Indeed, one of the aptamers, termed A15, was able to enter the endothelial cells and to reach the brain parenchyma. Although the target of A15 has not been addressed, the authors showed, for the first time, the possibility to adopt in vivo SELEX to recover aptamers that enter the mice brain upon peripheral delivery. 

Based on the work done in early 2000 by Shi et al., who exploited the RMT to transport cargoes through the BBB [97], DeRosa et al. now adapted a similar strategy to deliver a DBA payload into the brain [102] by using the TfR, which is highly expressed on endothelial cell surfaces and has long been reported to mediate transcytosis of therapeutic protein ligands through the BBB. Brain-penetrating bispecific therapeutic antibodies in which the anti-TfR antibody has been conjugated to a therapeutic antibody for the β-secretase enzyme have been reported showing that a lower binding affinity for TfR was more favorable than that of a high affinity antibody [83,84]. In their work, DeRosa et al. used a short DNA aptamer ligand for the TfR to induce RMT that was conjugated to the surface of PEGylated liposomes as carrier to drive DBA-payload across the BBB [102]. The approach adopted in which aptamers were used as both the transport mediator and payload was effective to promote the delivery and of the functional dopamine aptamer ligands from the peripheral injection site into the brain. Fernandez et al. have recently reported the generation of DNA aptamers capable to specifically bind and antagonize the Toll-like receptor 4 (TLR4) [103]. Developing drugs targeting TLR4 has become promising for stroke and multiple pathologies in which it is implicated [104]. TLR4 that is involved in pivotal processes, as inflammation and immunity, has been shown to mediate brain damage after stroke [105,106]. In order to obtain aptamers targeting TLR4, the authors adopted two parallel SELEX strategies: in the first, they used as complex target TLR4-expressing HEK293T cells (cell-SELEX); in the second, used as target the recombinant HIS-tagged TLR4 protein (protein-SELEX). They show that the truncated ApTLR#4FT specifically bind to and antagonize the TLR4 in vitro and in vivo. In a rat model, the ApTLR#4FT revealed to be protective against brain injury, in a TLR4-mediated manner, and showed to be able to cross the BBB and reach the ischemic mice brain region [103]. 

At difference of low grade tumors, high-grade gliomas are characterized by frequent alterations of the BBB that make the cerebrovascular barrier leaky and permeable to ONTs [107]. AS1411 is a G-quadruplex DNA aptamer that binds to nucleolin, a protein present on the surface of cancer cells [73]. Recently, Jiang et al. took advantage of AS1411 as targeting agent to address the delivery of cytotoxic agents through the cerebrovascular barrier. The aptamer was conjugated to PTX, a conventional drug inhibiting cancer cell mitotic activity that is effective for chemotherapeutic treatment of several cancer types including GBMs [108]. In order to enhance the solubility and reduce toxicity of PTX, the authors made use of a compound previously developed in their own laboratory, the poly (l-c-glutamyl-glutamine)-PTX (PGG-PTX) [109] to generate the dual-targeting AS1411-PGG-PTX nanoconjugate. AS1411-PGG-PTX demonstrated a good tumor penetration in intracranial U87 MG GBM-bearing nude mice, increasing the mouse median survival time. 

In a recent article, Macdonald et al. [31] addressed the uptake into the brain of a cancer therapeutic aptamer by conjugating it to an aptamer against the TfR as discussed above in Section 3. The generated bifunctional aptamer was able to successfully enter the brain by RMT and target tumors thus offering the proof of principle for the development of bispecific aptamers as potential therapeutic conjugates for brain tumors and metastasis.

## 8. Conclusions

In this review, we discussed some key and recent examples of aptamer-mediated delivery of nucleic acid and peptide-based therapeutics. The proposed examples underline the great potential of aptamers showing their exquisite targeting and specificity. In addition, they underscore how, thanks to their chemical nature, aptamers may be easily employed in the design of different flexible structures and modifications. This is an important advantage of the aptamer-based therapeutics that results in an easier production, in which complex manufacturing processes are avoided. 

Integrating the internalization properties and an endowed therapeutic potential, several aptamers can be also used for the development of multifunctional molecules in which multiple functions are combined to improve the selectivity and largely enhance the effectiveness of treatments. This can provide compelling rationale for new combination strategies. Furthermore, recent advances in the use of aptamers to specifically drive long ONT cargos have started to appear in literature, opening new path in the aptamer-delivery applicability. 

Despite the great promise, the road to clinical approval of aptamer-based conjugate still require several key steps regarding a better understanding of their fate within the target cells, their optimal formulation, PK/PD and toxicities profile. 

## References

[1] Foy J.W., Rittenhouse K., Modi M., Patel M. (2007). Local tolerance and systemic safety of pegaptanib sodium in the dog and rabbit. J. Ocul. Pharmacol. Ther..

[2] Keefe A.D., Pai S., Ellington A. (2010). Aptamers as therapeutics. Nat. Rev. Drug Discov..

[3] Nimjee S.M., White R.R., Becker R.C., Sullenger B.A. (2017). Aptamers as therapeutics. Annu. Rev. Pharmacol. Toxicol..

[4] Tuerk C., Gold L. (1990). Systematic evolution of ligands by exponential enrichment: RNA ligands to bacteriophage T4 DNA polymerase. Science.

[5] Ellington A.D., Szostak J.W. (1990). In vitro selection of RNA molecules that bind specific ligands. Nature.

[6] Ni S., Yao H., Wang L., Lu J., Jiang F., Lu A., Zhang G. (2017). Chemical modifications of nucleic acid aptamers for therapeutic purposes. Int. J. Mol. Sci..

[7] Hori S., Herrera A., Rossi J.J., Zhou J. (2018). Current advances in aptamers for cancer diagnosis and therapy. Cancers.

[8] Yoon S., Rossi J.J. (2018). Aptamers: Uptake mechanisms and intracellular applications. Adv. Drug Deliv. Rev..

[9] Tatiparti K., Sau S., Kashaw S.K., Iyer A.K. (2017). SiRNA delivery strategies: A comprehensive review of recent developments. Nanomaterials.

[10] Catuogno S., Esposito C.L., de Franciscis V. (2016). Aptamer-mediated targeted delivery of therapeutics: An update. Pharmaceuticals.

[11] Esposito C.L., Nuzzo S., Catuogno S., Romano S., de Nigris F., de Franciscis V. (2018). STAT3 gene silencing by aptamer-siRNA chimera as selective therapeutic for glioblastoma. Mol. Ther. Nucleic Acids.

[12] McNamara J.O., Andrechek E.R., Wang Y., Viles K.D., Rempel R.E., Gilboa E., Sullenger B.A., Giangrande P.H. (2006). Cell type-specific delivery of siRNAs with aptamer-siRNA chimeras. Nat. Biotechnol..

[13] Kruspe S., Giangrande P.H. (2017). Aptamer-siRNA Chimeras: Discovery, progress, and future prospects. Biomedicines.

[14] Zhou J., Neff C.P. (2013). Functional in vivo delivery of multiplexed anti-HIV-1 siRNAs via a chemically synthesized aptamer with a sticky bridge. Mol. Ther..

[15] Liu H.Y., Yu X., Liu H., Wu D., She J.X. (2016). Co-targeting EGFR and survivin with a bivalent aptamer-dual siRNA chimera effectively suppresses prostate cancer. Sci. Rep..

[16] Xue L., Maihle N.U., Yu X., Tang S.C., Liu H.Y. (2018). Synergistic targeting HER2 and EGFR with a bivalent aptamer-siRNA chimera efficiently inhibits HER2-positive tumor growth. Mol. Pharm..

[17] Jeong H., Lee S.H., Hwang Y., Yoo H., Jung H., Kim S.H., Mok H. (2017). Multivalent aptamer–RNA conjugates for simple and efficient delivery of doxorubicin/siRNA into multidrug-resistant cells. Macromol. Biosci..

[18] Alshaer W., Hillaireau H., Vergnaud J., Mura S., Deloménie C., Sauvage F., Ismail S., Fattal E. (2018). Aptamer-guided siRNA-loaded nanomedicines for systemic gene silencing in CD-44 expressing murine triple-negative breast cancer model. J. Control. Release.

[19] Takahashi M., Burnett J.C., Rossi J.J. (2015). Aptamer-siRNA chimeras for HIV. Adv. Exp. Med. Biol..

[20] Zhou J., Swiderski P., Li H., Zhang J., Neff C.P., Akkina R., Rossi J.J. (2009). Selection, characterization and application of new RNA HIV gp 120 aptamers for facile delivery of Dicer substrate siRNAs into HIV infected cells. Nucleic Acids Res..

[21] Neff C.P., Zhou J., Remling L., Kuruvilla J., Zhang J., Li H. (2011). An aptamer-siRNA chimera suppresses HIV-1 viral loads and protects from helper CD4(+) T cell decline in humanized mice. Sci. Transl. Med..

[22] Zhou J., Lazar D., Li H., Xia X., Satheesan S., Charlins P., O’Mealy D., Akkina R., Saayman S., Weinberg M.S. (2018). Receptor-targeted aptamer-siRNA conjugate-directed transcriptional regulation of HIV-1. Theranostics.

[23] Pang K.M., Castanotto D., Li H., Scherer L., Rossi J.J. (2018). Incorporation of aptamers in the terminal loop of shRNAs yields an effective and novel combinatorial targeting strategy. Nucleic Acids Res..

[24] Iaboni M., Russo V., Fontanella R., Roscigno G., Fiore D., Donnarumma E., Esposito C.L., Quintavalle C., Giangrande P.H., de Franciscis V. (2016). Aptamer-miRNA-212 conjugate sensitizes NSCLC cells to TRAIL. Mol. Ther. Nucleic Acids.

[25] Esposito C.L., Nuzzo S., Kumar S.A., Rienzo A., Lawrence C.L., Pallini R., Shaw L., Alder J.E., Ricci-Vitiani L., Catuogno S. (2016). A combined microRNA-based targeted therapeutic approach to eradicate glioblastoma stem-like cells. J. Control. Release.

[26] Russo V., Paciocco A., Affinito A., Roscigno G., Fiore D., Palma F., Galasso M., Volinia S., Fiorelli A., Esposito C.L. (2018). Aptamer-miR-34c conjugate affects cell proliferation of non-small cell lung cancer cells. Mol. Ther. Nucleic Acids.

[27] Gilboa E., McNamara J.N., Pastor F. (2013). Use of oligonucleotide aptamer ligands to modulate the function of immune receptors. Clin. Cancer Res..

[28] Khedri M., Rafatpanah H., Abnous K., Ramezani P., Ramezani M. (2015). Cancer immunotherapy via nucleic acid aptamers. Int. Immunopharmacol..

[29] Zheng J., Zhao S., Yu X., Huang S., Liu H.Y. (2017). Simultaneous targeting of CD44 and EpCAM with a bispecific aptamer effectively inhibits intraperitoneal ovarian cancer growth. Theranostics.

[30] Abnous K., Danesh N.M., Ramezani M., Yazdian-Robati R., Alibolandi M., Taghdisi S.M. (2017). A novel chemotherapy drug-free delivery system composed of three therapeutic aptamers for the treatment of prostate and breast cancers in vitro and in vivo. Nanomedicine.

[31] Macdonald J., Henri J., Goodman L., Xiang X., Duan W., Shigdar S. (2017). Development of a bifunctional aptamer targeting the transferrin receptor and epithelial cell adhesion molecule (EpCAM) for the treatment of brain cancer metastases. ACS Chem. Neurosci..

[32] Yu X., Ghamande S., Liu H., Xue L., Zhao S., Tan W., Zhao L., Tang S.-C., Wu D., Korkaya H. (2018). Targeting EGFR/HER2/HER3 with a three-in-one aptamer-siRNA chimera confers superior activity against HER2+ breast cancer. Mol. Ther. Nucleic Acids.

[33] Porcian D., Cardwell L., Tawiah K., Alam K., Lange M., Daniels M., Burke D. (2018). Modular cell-internalizing aptamer nanostructure enables targeted delivery of large functional RNAs in cancer cell lines. Nat. Commun..

[34] Filonov G.S., Moon J., Svensen N., Jaffrey S.R. (2014). Broccoli: Rapid selection of an RNA mimic of green fluorescent protein by fluorescence-based selection and directed evolution. J. Am. Chem. Soc..

[35] Paige J.S., Wu K.Y., Jaffrey S.R. (2011). RNA mimics of green fluorescent protein. Science.

[36] Maier K.E., Jangra R.K., Shieh K.R., Cureton K., Xiao H., Snapp E.L., Whelan S.P., Chandran K., Levy M. (2016). A new transferrin receptor aptamer inhibits new world hemorrhagic fever mammarenavirus entry. Mol. Ther. Nucleic Acids.

[37] Opazo F., Eiden L., Hansen L., Rohrbach F., Wengel J., Kjems J., Mayer G. (2015). Modular Assembly of cell-targeting devices based on an uncommon G-quadruplex aptamer. Mol. Ther. Nucleic Acids.

[38] Fosgerau K., Hoffmann T. (2015). Peptide therapeutics: Current status and future directions. Drug Discov. Today.

[39] Boohaker R.J., Lee M.W., Vishnubhotla P., Perez J.M., Khaled A.R. (2012). The use of therapeutic peptides to target and to kill cancer cells. Curr. Med. Chem..

[40] McClorey G., Banerjee S. (2018). Cell-penetrating peptides to enhance delivery of oligonucleotide-based therapeutics. Biomedicines.

[41] Bode S.A., Löwik D. (2017). Constrained cell penetrating peptides. Drug Discov. Today Technol..

[42] Xiang D., Zheng C., Zhou S.F., Qiao S., Tran P., Pu C., Li Y., Kong L., Kouzani A.Z., Lin J. (2015). Superior performance of aptamer in tumor penetration over antibody: Implication of aptamer-based theranostics in solid tumors. Theranostics.

[43] Romanelli A., Affinito A., Avitabile C., Catuogno S., Ceriotti P., Iaboni M., Modica J., Condorelli G., Catalucci D. (2018). An anti-PDGFRβ aptamer for selective delivery of small therapeutic peptide to cardiac cells. PLoS ONE.

[44] Rusconi F., Ceriotti P., Miragoli M., Carullo P., Salvarani N., Rocchetti M., Di Pasquale E., Rossi S., Tessari M., Caprari S. (2016). Peptidomimetic targeting of Cavβ2 overcomes dysregulation of the L-type calcium channel density and recovers cardiac function. Circulation.

[45] Chintalgattu V., Ai D., Langley R.R., Zhang J., Bankson J.A., Shih T.L. (2010). Cardiomyocyte PDGFR-β signaling is an essential component of the mouse cardiac response to load-induced stress. J. Clin. Investig..

[46] Rajabnejad S.H., Mokhtarzadeh A., Abnous K., Taghdisi S.M., Ramezani M., Razavi B.M. (2018). Targeted delivery of melittin to cancer cells by AS1411 anti-nucleolin aptamer. Drug Dev. Ind. Pharm..

[47] Smith J.D., Cardwell L.N., Porciani D., Nguyen J.A., Zhang R., Gallazzi F., Tata R.R., Burke D.H., Daniels M.A., Ulery B.D. (2018). Aptamer-displaying peptide amphiphile micelles as a cell-targeted delivery vehicle of peptide cargoes. Phys. Biol..

[48] Charbgoo F., Alibolandi M., Taghdisi S.M., Abnous K., Soltani F., Ramezani M. (2018). MUC1 aptamer-targeted DNA micelles for dual tumor therapy using doxorubicin and KLA peptide. Nanomedicine.

[49] Xiaoting J., Haoyuan L., Guo J., Ding C., Luo X. (2018). A DNA nanotube–peptide biocomplex for mRNA detection and its application in cancer diagnosis and targeted therapy. Chemistry.

[50] Heo K., Min S.W., Sung H.J., Kim H.G., Kim H.J., Kim Y.H., Choi B.K., Han S., Chung S., Lee E.S. (2016). An aptamer-antibody complex (oligobody) as a novel delivery platform for targeted cancer therapies. J. Control. Release.

[51] Hsu P.D., Lander E.S., Zhang F. (2014). Development and applications of CRISP-Cas9 for genome engineering. Cell.

[52] Zhen S., Hua L., Takahashi Y., Narita S., Liu Y.H., Li Y. (2014). In vitro and in vivo growth suppression of human papillomavirus 16-positive cervical cancer cells by CRISP/Cas9. Biochem. Biophys. Res. Commun..

[53] Cheong T.C., Compagno M., Chiarle R. (2016). Editing of mouse and human immunoglobulin genes by CRISP-Cas9 system. Nat. Commun..

[54] Li L., He Z.Y., Wei X.W., Gao G.P., Wei Y.Q. (2015). Challenges in CRISPR/Cas9 delivery: Potential roles of nonviral vectors. Hum. Gene Ther..

[55] Zhou J., Bobbin M.I., Burnett J.C., Rossi J.J. (2012). Current progress of RNA aptamer-based therapeutics. Front. Genet..

[56] Shalem O., Sanjana N., Zhang F. (2015). High-throughput functional genomics using CRISPR-Cas9. Nat. Rev. Genet..

[57] Liu J., Shui S.L. (2016). Delivery methods for site-specific nucleases: Achieving the full potential of therapeutic gene editing. J. Control. Release.

[58] David R.M., Doherty A.T. (2017). Viral vectors: The road to reducing genotoxicity. Toxicol. Sci..

[59] Yang Y., Wang L., Bell P., McMenamin D., He Z.Y., White J., Yu H., Xu C., Morizono H., Musunuru K. (2016). A dual AAV system enables the Cas9-mediated correction of a metabolic liver disease in newborn mice. Nat. Biotechnol..

[60] Senís E., Fatouros C., Große S., Wiedtke E., Niopek D., Mueller A.K., Börner K., Grimm D. (2014). CRISPR/Cas9-mediated genome engineering: An adeno-associated viral (AAV) vector toolbox. Biotechnol. J..

[61] Karnan S., Ota A., Konishi Y., Wahiduzzaman M., Hosokawa Y., Konishi H. (2016). Improved methods of AAV-mediated gene targeting for human cell lines using ribosome-skipping 2A peptide. Nucleic Acids Res..

[62] Maggio I., Holkers M., Liu J., Janssen J.M., Chen X., Goncalves M.A. (2015). Adenoviral Vector Delivery of RNA-guided CRISPR/Cas9 nuclease complexes induces targeted mutagenesis in a diverse array of human cells. Sci. Rep..

[63] Maggio I., Liu J., Janssen J.M., Chen X., Goncalves M.A. (2016). Adenoviral vectors encoding CRISPR/cas9 multiplexes rescue dystrophin synthesis in unselected populations of DMD muscle cells. Sci. Rep..

[64] Gong H., Liu M., Klomp J., Merrill B.J., Rehman J., Malik A.B. (2017). Method for dual viral vector mediated CRISPR-Cas9 gene disruption in primary human endothelial cells. Sci. Rep..

[65] Koike-Yusa H., Li Y., Tan E.P., Velasco-Herrera M.D., Yusa K. (2014). Genome-wide recessive genetic screening in mammalian cells with a lentiviral CRISPR-guide RNA library. Nat. Biotechnol..

[66] Suzuki K., Tsunekawa Y., Hernandez-Benitez R., Wu J., Zhu J., Kim E.J., Hatanaka F., Yamamoto M., Araoka T., Li Z. (2016). In vivo genome editing via CRISPR/Cas9 mediated homology-independent targeted integration. Nature.

[67] Kang Y.K., Kwon K., Ryu J.S., Lee H.N., Park C., Chung H.J. (2017). Nonviral genome editing based on a polymer-derivatized CRISPR nanocomplex for targeting bacterial pathogens and antibiotic resistance. Bioconjug. Chem..

[68] Cramer-Morales K., Heer C.D., Mapuskar K.A., Domann F.E. (2015). *SOD2* targeted gene editing by CRISPR/Cas9 yields human cells devoid of MnSOD. Free Radic. Biol. Med..

[69] Radis-Baptista G., Campelo I.S., Morlighem J.R.L., Melo L.M., Freitas V.J.F. (2017). Cell-penetrating peptides (CPP): From delivery of nucleic acids and antigens to transduction of engineered nucleases for application in transgenesis. J. Biotechnol..

[70] Zhou J., Li H., Zaia J., Rossi J.J. (2008). Novel dual inhibitory function aptamer-siRNA delivery system for HIV-1 therapy. Mol. Ther..

[71] Zhen S., Takahashi Y., Narita S., Yang Y.C., Li X. (2017). Targeted delivery of CRISPR/Cas9 to prostate cancer by modified gRNA using a flexible aptamer-cationic liposome. Oncotarget.

[72] Liang C., Li F., Wang L., Zhang Z.-K., Wang C., He B., Li J., Chen Z., Shaikh A.B., Liu J. (2017). Tumor cell-targeted delivery of CRISPR/Cas9 by aptamer-functionalized lipopolymer for therapeutic genome editing of VEGFA in osteosarcoma. Biomaterials.

[73] Bates P.J., Laber D.A., Miller D.M., Thomas S.D., Trent J.O. (2009). Discovery and development of the G-rich oligonucleotide AS1411 as a novel treatment for cancer. Exp. Mol. Pathol..

[74] Dam D.H.M., Lee J.H., Sisco P.N., Co D.T., Zhang M., Wasielewski M.R., Odom T.W. (2012). Direct observation of nanoparticle-cancer cell nucleus interactions. ACS Nano.

[75] Wang Y., Zhang T., Kwiatkowski N., Abraham B.J., Lee T.L., Xie S., Yuzugullu H., Von T., Li H., Lin Z. (2015). CDK7-dependent transcriptional addiction in triple-negative breast cancer. Cell.

[76] Feng Y., Sassi S., Shen J.K., Yang X., Gao Y., Osaka E., Zhang J., Yang S., Yang C., Mankin H.J. (2015). Targeting CDK11 in osteosarcoma cells using the CRISPR-Cas9 system. J. Orthop. Res..

[77] Dever D.P., Bak R.O., Reinisch A., Camarena J., Washington G., Nicolas C.E., Pavel-Dinu M., Saxena N., Wilkens A.B., Mantri S. (2018). CRISPR/Cas9 β-globin gene targeting in human hematopoietic stem cells. Nature.

[78] DeWitt M.A., Magis W., Bray N.L., Wang T., Berman J.R., Urbinati F., Heo S.J., Mitros T., Muñoz D.P., Boffelli D. (2016). Selection-free genome editing of the sickle mutation in human adult hematopoietic stem/progenitor cells. Sci. Transl. Med..

[79] Carlson-Stevermer J., Abdeen A.A., Kohlenberg L., Goedland M., Molugu K., Lou M., Saha K. (2017). Assembly of CRISPR ribonucleoproteins with biotinylated oligonucleotides via an RNA aptamer for precise gene editing. Nat. Commun..

[80] Lin Y., Qiu Q., Gill S.C., Jayasena S.D. (1994). Modified RNA sequence pools for in vitro selection. Nucleic Acids Res..

[81] Ruckman J., Green L.S., Beeson J., Waugh S., Gillette W.L., Henninger D.D., Claesson-Welsh L., Janjic N. (1998). 2′-Fluoropyrimidine RNA-based aptamers to the 165-amino acid form of vascular endothelial growth factor (VEGF165). Inhibition of receptor binding and VEGF-induced vascular permeability through interactions requiring the exon 7-encoded domain. J. Biol. Chem..

[82] Burmeister P.E., Lewis S.D., Silva R.F., Preiss J.R., Horwitz L.R., Pendergrast P.S., McCauley T.G., Kurz J.C., Epstein D.M., Wilson C. (2005). Direct in vitro selection of a 2′-O-methyl aptamer to VEGF. Chem. Biol..

[83] Gold L., Ayers D., Bertino J., Bock C., Bock A., Brody E.N., Carter J., Dalby A.B., Eaton B.E., Fitzwater T. (2010). Aptamer-based multiplexed proteomic technology for biomarker discovery. PLoS ONE.

[84] Vaught J.D., Bock C., Carter J., Fitzwater T., Otis M., Schneider D., Rolando J., Waugh S., Wilcox S.K., Eaton B.E. (2010). Expanding the chemistry of DNA for in vitro selection. J. Am. Chem. Soc..

[85] Maasch C., Buchner K., Eulberg D., Vonhoff S., Klussmann S. (2008). Physicochemical stability of NOX-E36, a 40mer L-RNA (Spiegelmer) for therapeutic applications. Nucleic Acids Symp. Ser. (Oxf.).

[86] Rusconi C.P., Roberts J.D., Pitoc G.A., Nimjee S.M., White R.R., Quick G., Scardino E., Fay W.P., Sullenger B.A. (2004). Antidote-mediated control of an anticoagulant aptamer in vivo. Nat. Biotechnol..

[87] Lee C.H., Lee S.-H., Kim J.H., Noh Y.-H., Noh G.-J., Lee S.-W. (2015). Pharmacokinetics of a cholesterol-conjugated aptamer against the Hepatitis C virus (HCV) NS5B protein. Mol. Ther. Nucleic Acids.

[88] Dougan H., Lyster D.M., Vo C.V., Stafford A., Weitz J.I., Hobbs J.B. (2000). Extending the lifetime of anticoagulant oligodeoxynucleotide aptamers in blood. Nucl. Med. Biol..

[89] Willis M.C., Collins B.D., Zhang T., Green L.S., Sebesta D.P., Bell C., Kellogg E., Gill S.C., Magallanez A., Knauer S. (1998). Liposome-anchored vascular endothelial growth factor aptamers. Bioconjug. Chem..

[90] Zhou J., Soontornworajit B., Martin J., Sullenger B.A., Gilboa E., Wang Y. (2009). A hybrid DNA aptamer-dendrimer nanomaterial for targeted cell labeling. Macromol. Biosci..

[91] Chen L., Rashid F., Shah A., Awan H.M., Wu M., Liu A., Wang J., Zhu T., Luo Z., Shan G. (2015). The isolation of an RNA aptamer targeting to p53 protein with single amino acid mutation. Proc. Natl. Acad. Sci USA.

[92] Drolet D.W., Nelson J., Tucker C.E., Zack P.M., Nixon K., Bolin R., Judkins M.B., Farmer J.A., Wolf J.L., Gill S.C. (2000). Pharmacokinetics and safety of an anti-vascular endothelial growth factor aptamer (NX1838) following injection into the vitreous humor of rhesus monkeys. Pharm. Res..

[93] Tucker C.E., Chen L.S., Judkins M.B., Farmer J.A., Gill S.C., Drolet D.W. (1999). Detection and plasma pharmacokinetics of an anti-vascular endothelial growth factor oligonucleotide-aptamer (NX1838) in rhesus monkeys. J. Chromatogr. B. Biomed. Sci. Appl..

[94] Ganson N.J., Povsic T.J., Sullenger B.A., Alexander J.H., Zelenkofske S.L., Sailstad J.M., Rusconi C.P., Hershfield M.S. (2016). Pre-existing anti-polyethylene glycol antibody linked to first-exposure allergic reactions to pegnivacogin, a PEGylated RNA aptamer. J. Allergy Clin. Immunol..

[95] Waring M.J. (2010). Lipophilicity in drug discovery. Expert Opin. Drug Discov..

[96] Pavan B., Dalpiaz A., Ciliberti N., Biondi C., Manfredini S., Vertuani S. (2008). Progress in drug delivery to the central nervous system by the prodrug approach. Molecules.

[97] Shi N., Pardridge W.M. (2000). Noninvasive gene targeting to the brain. Proc. Natl. Acad. Sci. USA.

[98] Pardridge W.M. (2007). Blood-brain barrier delivery of protein in non-viral gene therapeutics with molecular Trojan horses. J. Control. Release.

[99] Cerchia L., de Franciscis V. (2010). Targeting cancer cells with nucleic acid aptamers. Trends Biotechnol..

[100] Holahan M.R., Madular D., McConnell E.M., Walsh R., DeRosa M.C. (2011). Intra-accumbens injection of a dopamine aptamer abates MK-801-induced cognitive dysfunction in a model of schizophrenia. PLoS ONE.

[101] Cheng C., Chen Y.H., Lennox K.A., Behlke M.A., Davidson B.L. (2013). In vivo SELEX for Identification of Brain-penetrating Aptamers. Mol. Ther. Nucleic Acids.

[102] McConnell E.M., Ventura K., Dwyer Z., Hunt V., Koudrina A., Holahan M.R., De Rosa M.C. (2018). In vivo use of a multi-DNA aptamer-based payload/targeting system to study dopamine dysregulation in the central nervous system. ACS Chem. Neurosci..

[103] Fernández G., Moraga A., Cuartero M., García-Culebras A., Peña-Martínez C., Pradillo J.M., Hernández-Jiménez M., Sacristán S., Ayuso M., Gonzalo-Gobernado R. (2018). TLR4-binding DNA aptamers show a protective effect against acute stroke in animal models. Mol. Ther..

[104] Hennessy E.J., Parker A.E., O’Neill L.A. (2010). Targeting Toll-like receptors: Emerging therapeutics?. Nat. Rev. Drug Discov..

[105] Caso J.R., Pradillo J.M., Hurtado O., Leza J.C., Moro M.A., Lizasoain I. (2008). Toll-like receptor 4 is involved in subacute stress-induced neuroinflammation and in the worsening of experimental stroke. Stroke.

[106] Hamanaka J., Hara H. (2011). Involvement of Toll-like receptors in ischemia-induced neuronal damage. Cent. Nerv. Syst. Agents Med. Chem..

[107] Van Tellingen O., Yetkin-Arik B., de Gooijer M.C., Wesseling P., Wurdinger T., de Vries H.E. (2015). Overcoming the blood-brain tumor barrier for effective glioblastoma treatment. Drug Resist. Updates.

[108] Luo Z., Yan Z., Jin K., Pang Q., Jiang T., Lu H., Liu X., Pang Z., Yu L., Jiang X. (2017). Precise glioblastoma targeting by AS1411 aptamer-functionalized poly (l-γ-glutamylglutamine)-paclitaxel nanoconjugates. J. Colloid Interface Sci..

[109] Yang D., Van S., Jiang X., Yu L. (2011). Novel free paclitaxel-loaded poly(L-γ-glutamylglutamine)-paclitaxel nanoparticles. Int. J. Nanomed..

